# DNA barcoding of Japanese earwig species (Insecta, Dermaptera), with sequence diversity analyses of three species of Anisolabididae

**DOI:** 10.3897/BDJ.11.e107001

**Published:** 2023-09-27

**Authors:** Yoshitaka Kamimura, Masaru Nishikawa, Junsuke Yamasako

**Affiliations:** 1 Keio University, Yokohama, Japan Keio University Yokohama Japan; 2 Ehime University, Matsuyama, Japan Ehime University Matsuyama Japan; 3 National Agriculture and Food Research Organization, Tsukuba, Japan National Agriculture and Food Research Organization Tsukuba Japan

**Keywords:** Anisolabididae, Chelisochidae, Diplatyidae, East Asia, Forficulidae, Labiduridae, Polyneoptera, Pygidicranidae, semi-synanthropic species, Spongiphoridae, within-species sequence diversity

## Abstract

Dermaptera is a polyneopteran insect order that includes more than 2,000 described species, commonly known as earwigs, that mainly inhabit tropical, subtropical and warm temperate regions. Although 40 species have been found in Japan, their distribution and habitat preferences have remained ambiguous due to sample misidentification, particularly amongst immature specimens. To overcome this problem, we sequenced and analysed the DNA barcoding region of the mitochondrial cytochrome oxidase I gene (*cox1*) of dermapteran species recorded from Japan. Including publicly available data, 72.5% of known Japanese dermapteran species were subjected to molecular identification. We extensively sampled three wingless species of subfamily Anisolabidinae (Anisolabididae): *Anisolabismaritima*, *Anisolabellamarginalis* and *Euborelliapallipes*. Although these species exhibit similar habitat preferences as semi-synanthropes, *A.maritima*, a cosmopolitan species with the highest affinity to seashore, had significantly higher sequence diversity than the latter two species, which are considered endemic to East Asia. A similar trend was observed for (at least partly) winged cosmopolitan species of other families. Introgression with the congener *Anisolabisseirokui* is also suggested for *A.maritima*. Possible causes of the varying levels of sequence diversity are discussed.

## Introduction

DNA barcoding, i.e. the determination of species-specific sequences of highly-variable DNA regions, is a powerful tool for species diagnosis and for discriminating closely-related species, particularly when the availability of morphological characters and/or competency of the examining researchers are limited (Hebert et al. 2003b, Armstrong and Ball 2005, Antil et al. 2023). However, its effectiveness depends on the species coverage of sequence databases. More than 2,000 species of order Dermaptera (Insecta), which are commonly known as earwigs (in English) or Hasami-mushi (in Japanese), have been reported to date (Zhang 2013, Hopkins et al. 2018). Covering environmental and climatic heterogeneity from subboreal to subtropical, 40 earwig species (i.e. ca. 2% of the worldwide species diversity) have been reported from Japan (Table 1), which represents only 0.28% of the global terrestrial area (Nishikawa 2016, Nishikawa and Naka 2019, Nishikawa 2020a, Kamimura et al. 2023). As only a few researchers study the taxonomy of this comparatively small insect order, many dermapteran samples have been misidentified by amateur scientists and non-specialised researchers, causing serious confusion and delays in evaluating the dermapteran diversity of Japan (Nishikawa 2005, Nishikawa 2013, Nishikawa 2016, Nishikawa 2020a). This is particularly true for nymphal samples, which lack many of the characteristics necessary for species diagnosis (Nishikawa 2007). For example, a nymphal dermapteran from Japan was even misidentified as an adult isopteran (Hagen 1868a, Hagen 1868b, Snyder 1916: see Kočárek and Hu (2023) for a similar confusion with Zoraptera in Taiwan).

Several earwig species, including many members of the subfamily Anisolabidinae (Anisolabididae), are completely wingless in all life stages or are flightless because they lack hind-wings possessing only (vestigial or complete) tegmina (Sakai 1987, Steinmann 1989a, Srivastava 2003), limiting their dispersal ability. Nevertheless, some wingless species, such as *Anisolabismaritima* (Bonelli, 1832) and *Euborelliaannulipes* (Lucas, 1847) occur almost worldwide including many small continental and oceanic islands (e.g. Brindle (1969a), Brindle (1970), Kevan and Vickery (1997)), whereas others are distributed only locally. For example, both *Gonolabismichikoae* Nishikawa, 2021 and *G.miyatakei* Nishikawa, 2021 are considered endemic to Amami-Oshima, an island (712.35 km²) in subtropical Japan (Nishikawa 2021). *Euborelliapallipes* (Shiraki, 1906) specimens have been confused with congeners, such as *Euborelliaannulata* (Fabricius, 1793) (brachypterous species) and *Euborelliaplebeja* (Dohrn, 1863) (winged species), which are more widely distributed (Kamimura et al. 2023). This species has sometimes been reported as *E.pallipes* (Shiraki, 1905); however, the original description was published in 1906 (Shiraki 1906). *Euborelliapallipes* is brachypterous and presently considered to occur only in East Asia (Kamimura et al. 2023), as is *Anisolabellamarginalis* (Dohrn, 1864), which is a completely apterous species with credible records only from the Korean Peninsula and Japan (Nishikawa 2020a).

To overcome the problem of dermapteran species misidentification in Japan, we applied DNA barcoding to the largest possible number of dermapteran species recorded from Japan. Due to the high evolutionary rate, DNA barcode analyses can provide insights into the causes and consequences of within-species genetic diversity, in relation to habitat, life history and distribution. In this study, we performed detailed analyses of three flightless species of Anisolabidae, *Anisolabismaritima* (= *A.maritima*), *Anisolabellamarginalis* (= *Al.marginalis*) and *Euborelliapallipes*, based on extensive sampling in Japan. Their sequence diversity was also compared to that of at least partly-winged earwig species, such as *Labiaminor* (Linnaeus, 1758) (= *L.minor*) and *Labidurariparia* (Pallas, 1773) (= *Ld.riparia*).

## Materials and methods

### Sample collection, PCR amplification and sequencing

Dermapteran samples were collected by hand-sorting (except for *L.minor*, the foundress of the laboratory population was collected in a light trap) from various localities in Japan (Table 2). Total genomic DNA was extracted from fresh, ethanol-preserved or, in a few cases, dried samples using a DNeasy Blood & Tissue Kit (Qiagen, Hilden, Germany), according to the manufacturer’s instructions. Depending on the size of the specimens, one to five legs were used for DNA extraction. Adult male samples were used whenever available. Polymerase chain reaction (PCR) amplification of the mitochondrial cytochrome c oxidase subunit I (COX1) gene region (658 bp), which is widely used in the DNA barcoding of earwigs (Matzke and Kočárek 2015, Stuart et al. 2019, Kalaentzis et al. 2021, Kočárek and Wahab 2021, Kamimura et al. 2023) and other invertebrates (Folmer et al. 1994) was performed using a T100^TM^ thermal cycler (Bio-Rad Laboratories, Hercules, CA, USA) and primers LCO1490 and HCO2198 (Folmer et al. 1994). PCR reactions were conducted in a 20 μl volume containing 1 μl each primer (10 μM), 10 μl 2×PCR buffer, 4 μl dNTPs (2 mM each), 0.4 μl KOD FX Neo DNA polymerase (1.0 unit/μl; Toyobo, Osaka, Japan) and 1 μl genomic DNA (or a total volume of 5 μl for some samples). The PCR temperature profile consisted of 2 min at 94°C, followed by 35 cycles of 15 s at 94°C, 15 s at 51°C and 15 s at 72°C, followed by a final extension for 6 min at 72°C. For several samples, for which the primer set did not work, another set of primers (SKCOI-7 and KSCOI-2; Su et al. (2004)) was used to obtain PCR products: products by these primers largely overlapped the LCO1490–HCO2198 region. PCR reactions were conducted in a 10 or 20 μl volume and the PCR temperature profile consisted of 2 min at 94°C, followed by 35–40 cycles of 10 s at 98°C, 30 s at 50°C and 60 s at 68°C, followed by a final extension of 8 min at 68°C. Sequencing was performed by Eurofins Genomics (Tokyo, Japan) or FASMAC (Kanagawa, Japan: for some samples). The chromatograms were checked visually and edited manually where appropriate. The sequences will be deposited in the DNA Data Bank of Japan (DDBJ), European Nucleotide Archive (ENA) and GenBank. Voucher specimens with codes starting with 2021BC were deposited in the personal collection of YK (Keio University, Japan) and those with codes starting with NARO-Dermaptera were deposited in the Insect Museum of the National Agriculture and Food Research Organization (NARO), Japan.

### Sequence diversity analysis

Multiple sequence alignments were conducted using the ClustalW programme (Thompson et al. 2003) implemented in MEGA11 (Tamura et al. 2021) with the default settings. Due to the high evolutionary rates of DNA, it is generally difficult to estimate phylogenetic relationships amongst dermapteran genera, based solely on sequences of the region (Kamimura et al. 2023). Accordingly, the Maximum Likelihood (ML) tree was estimated to analyse and visualise relationships amongst closely-related species (usually congeners) and sequence diversity within species. Taking into account the uncertainty in sample identification, particularly that of Anisolabididae, we did not include sequence data that are available in the Barcode of Life Data Systems (BOLD) public repository (https://v3.boldsystems.org/) for non-Japanese dermapteran species. For the following species recorded from Japan, we included all sequence data for samples from foreign localities available in BOLD (accessed 28 March 2023) and those reported in Kamimura et al. (2023) for Malaysian populations: *A.maritima*, *E.annulipes*, *Ld.riparia*, *Paralabellulacurvicauda* (Motschulsky, 1863) and *L.minor* and *Proreussimulans* (Stål, 1860).

The ML analysis and calculation of intraspecific genetic distances (*p*-distances) were performed using MEGA11. For ML analysis, the optimal nucleotide substitution model (general time-reversible model [GTR]+G+I) was determined using the Bayesian Information Criterion (BIC) and the default search algorithms and settings, which included a discrete Gamma distribution (+G) with five rate categories and an evolutionarily invariable fraction of sites (+I). As no non-dermapteran samples were added as outgroups, the resultant barcode tree was rooted by a clade of the infraorder Protodermaptera.

### Sequence diversity comparison amongst species

For *A.maritima*, *Al.marginalis* and *E.pallipes*, we analysed samples from multiple localities of Japan. To statistically compare within-species sequence diversity amongst these species, we selected 14 localities (or multiple localities within 15 km of each other), from which all three species were obtained (Table 2). For localities with multiple samples sequenced (*A.maritima* of Naruto, Tokushima Pref. and Shirako, Chiba Pref.), a single sample was randomly chosen for analysis because the sequences in each locality were identical (see Results). We calculated the mean within-species *p*-distance and its standard error for each species using MEGA11. The latter was estimated by bootstrapping the sequences 1,000 times. To test the differences in the mean *p*-distance between species, we performed a permutation test (Manly and Alberto 2021). In this analysis, *p*-distances were randomly shuffled amongst three species for each of 91 combinations amongst the 14 localities, using a personal script written in Python v.3.8.3 implemented with the *Numpy* v.1.23.1 package. Interspecific differences in the mean *p*-distance were compared amongst the 10,000-replicate permutated data (9,999 shuffled values plus one actual value) and actual values to determine the level of significance.

## Results and discussion

### PCR amplification and interspecific relationships

The universal primer set, LCO1490 and HCO2198, worked well for many dermapteran species, resulting in the amplification of 658 bp of the 5’ DNA-barcoding region of the mitochondrial *cox1* gene (for LC767869, LC767870 and LC767871, 11–135 bases of 3’-end could not be determined). Exceptions included *Challiaimamurai* Nishikawa, 2006 (Pygidicranidae: partly; LC767872), *Euborelliaannulipes* (Anisolabididae: all; see Kamimura et al. (2023)) and *Proreussimulans* (Chelisochidae: all; LC767873). For these samples, an alternate primer set (SKCOI-7 and KSCOI-2) was successfully applied to amplify products largely overlapping with the LCO1490-HCO2198 region. Interestingly, no consistent taxonomic bias was observed in species for which the LCO1490-HCO2198 primer set failed to work, suggesting that one or both of these primers did not match the annealing site due to the extremely high rate of molecular evolution. As a result of this work, data for this region are now available for at least one sample of 29 of 40 (72.5%) species of earwigs recorded from Japan (Table 1).

**Figure 1.**
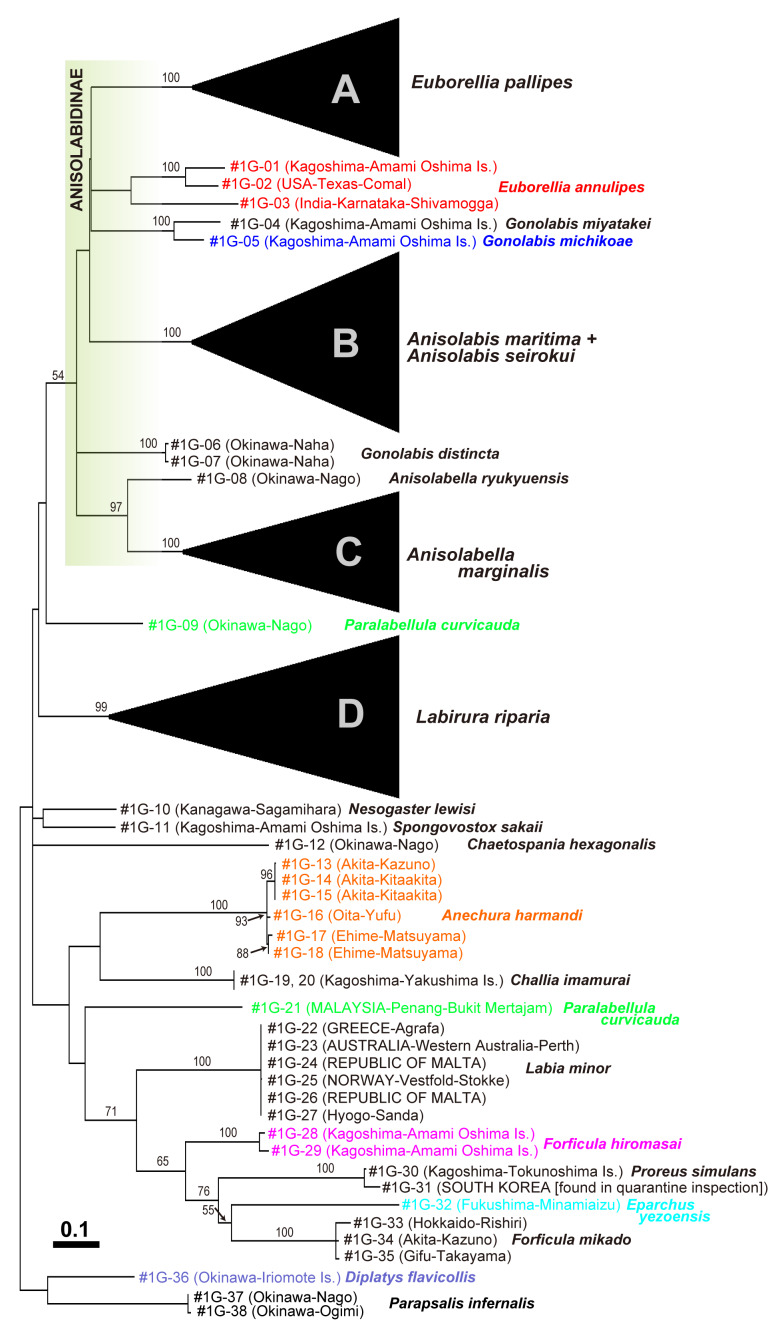
Maximum Likelihood barcode tree constructed from mitochondrial cytochrome oxidase I gene (*cox1*) sequence data. Branch numbers indicate bootstrap values (% in 500 replicates; only values ≥ 50% are shown). Four clades (all supported with ≥ 99% bootstrap values) containing more than 20 samples (A-D) are compressed. Localities are indicated in parentheses in the format “Prefecture-City (or Island)” for samples collected in Japan. For large cities with multiple wards (or small towns within a district), the name of the ward (or town) replaces the city name, with the city or district name in brackets, where necessary. For foreign samples, the country is indicated in capital letters. A unique sample code precedes the locality. Detailed sample descriptions are provided in Table 2.

The extremely high rate of molecular evolution makes the sequenced region useful for the diagnosis and delimitation of closely-related species (Hebert et al. 2003a, Hebert et al. 2003b, Hebert et al. 2004). However, estimating deeper phylogenies (usually relationships amongst genera or deeper) is usually difficult, based solely on this region due to base substitution saturation (Folmer et al. 1994, Talavera et al. 2021) and it is true for Dermaptera (Kamimura et al. 2023). In addition, the phylogenetic relationships estimated, based on only markers of mitochondrial DNA, can be distorted by the effects of hybridisation or introgression (Funk and Omland 2003, Hebert et al. 2003b). Accordingly, interspecific relationships were not resolved (< 50% bootstrap support) in the ML barcode tree (Fig. 1; Table 3). Although only a few representatives were sampled for each genus, monophyly was indicated for the subfamily Anisolabidinae (Anisolabididae: 54%), the genera *Anisolabis* (100%) and *Anisolabella* (97%), two species of *Gonolabis* endemic to Amami-Oshima (*G.michikoae* + *G.miyatakei*: 100%) and a clade including *Forficulamikado* Burr, 1904 + *Forficulahiromasai* Nishikawa, 1970 + *Eparchusyezoensis* (Matsumura et Shiraki, 1905) + *P.simulans* (71%). In the latter, relationships (*L.minor*, (*F.hiromasai*, (*P.simulans*, (*E.yezoensis*, *F.mikado*)))) were also suggested with weak support (55–76%) (Fig. 1).

**Figure 2.**
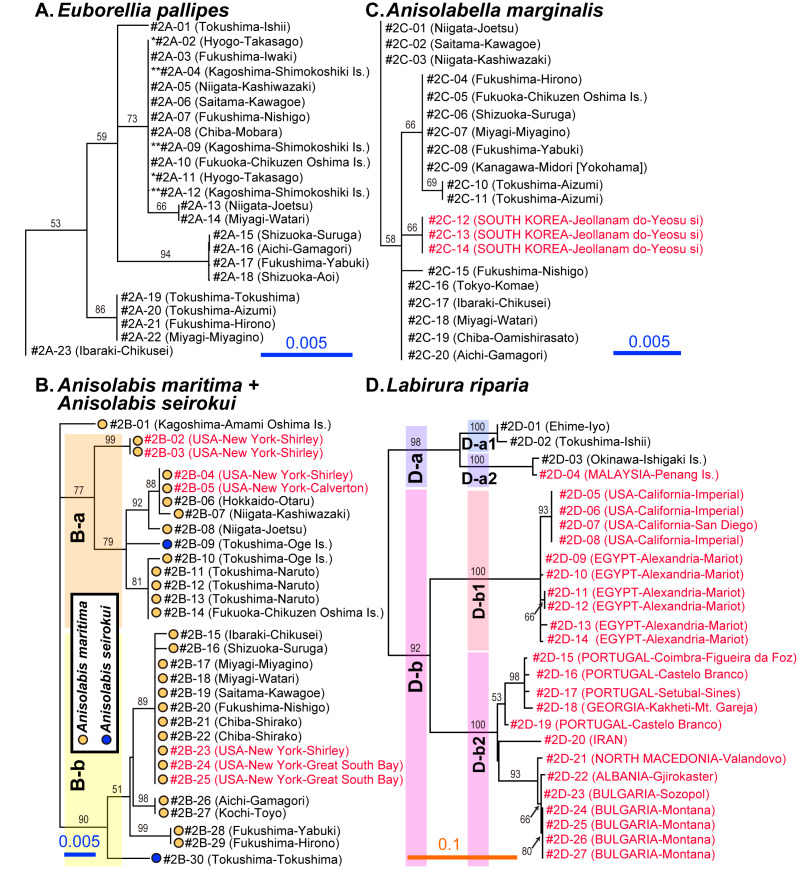
Expanded cladograms of Clade A–D (Fig. 1). Unique sample codes, localities and bootstrap values are indicated as in Fig. 1. Samples collected in Japan and foreign countries are indicated in black and red letters, respectively. Samples with the same numbers of asterisks were derived from a single, wild-caught female in **A**.

Interestingly, the monophyletic *Anisolabis* includes two major groups, clade B-a and B-b (Fig. 2B), each of which was supported with > 75% bootstrap value. Four samples of *A.maritima* from New York, USA, were placed within Clade B-a, two of which formed a subclade (99% support), whereas three others were placed in Clade B-b. Furthermore, two samples of *A.seirokui* from Tokushima Prefecture, Japan, were completely separated into the two major clades (blue circles, Fig. 2B), rendering *A.maritima* (orange circles, Fig. 2B) paraphyletic. In the samples of *A.maritima* from Japan, an individual from the southernmost locality (Amami-Oshima) was included in neither clade B-a nor B-b. The others were divided into the two clades, indicating geographical differentiation between Shikoku (partial) and the Sea of Japan coast of Honshu + Hokkaido + Kyushu (Clade B-a) and the Pacific coast of Honshu + Shikoku (Clade B-b). The maximum *p*-distance amongst the studied samples of *A.maritima* was 2.7%, slightly exceeding 2.0%, which is often used to delimit insect species boundaries (Cognato 2006). However, some species exhibit remarkably high intraspecific diversity (> 5%) in the mitochondrial COI DNA sequences (up to 26.0%: Cognato (2006)), warranting further study of reproductive isolation amongst the *Anisolabis* clades.

*Anisolabismaritima* is a cosmopolitan species that occurs in all faunal regions, but is usually restricted to warm temperate, subtropical and tropical areas (Brindle 1969a, Sakai 1987, Steinmann 1989a, Srivastava 2003). As the species epithet suggests, this species is especially abundant under seashore debris in temperate Japan (Matsumura 1935, Tobiyama 1985, Kamimura 2005) and foreign localities (Bennet 1904, Caudell 1913, Hebard 1917, Hincks 1947, Guppy 1950, Brindle 1969a). By contrast, *A.seirokui*, which has been recorded from Japan, the Korean Peninsula (South Korea) and Taiwan, is also a maritime species, but usually found in rocky seashores, rocky reefs or artificial marine breakwaters (Nishikawa 2016, Nishikawa 2020a). The much more slender body shape of *A.seirokui* with its less asymmetrical, thinner male forceps (Nishikawa 2008, Nishikawa 2016) may represent an adaptation to their cryptic life in rocky environments with many narrow crevices, suggesting ecological differentiation with *A.maritima*. The observed polyphyly of *A.seirokui*, which is nested in the clades of *A.maritima*, may reflect incomplete lineage sorting after divergence of their ancestors or the results of past or present introgressions (gene flow due to hybridisation) between these incipient species (Pollard et al. 2006, Degnan and Rosenberg 2009, Fontaine et al. 2015, Mallet et al. 2016). Further sampling (especially specimens of *A.seirokui*) and analyses of multiple genes, including nuclear genes, are needed to resolve these issues.

### Intraspecific diversity

In contrast to the low power associated with constructing deeper phylogenies, the monophyly of each species with multiple samples was strongly supported with a ~ 100% bootstrap value, except for *E.annulipes*, *P.curvicauda* and the possible paraphyly of *A.maritima* (polyphyly of *A.seirokui*) (Fig. 1). For *E.annulipes*, the sequence of an Indian sample showed ca. 16–17% divergence from samples from USA and Japan, which showed 10% difference, but are undoubtedly closely related to each other (100% support).

**Figure 3.**
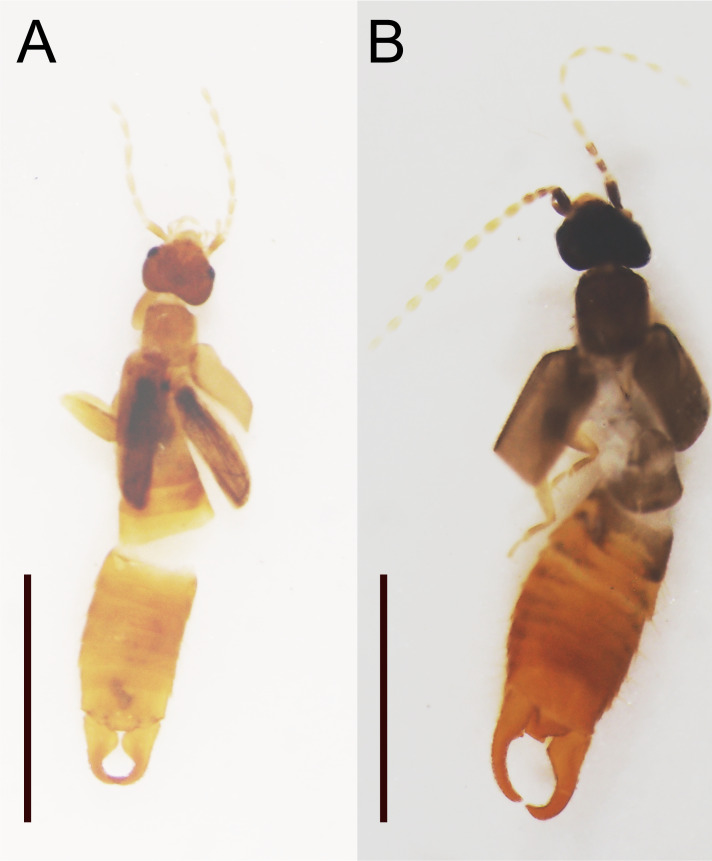
Voucher specimens (preserved in ethanol) of *Paralabellulacurvicauda*. **A** 2021BCDNA40 from Malaysia (Kamimura et al. 2023); **B** 2021BCDNA57 from Japan. Scale bars: 2 mm.

The most notable example is the spongiphorid *P.curvicauda*: the sequence of a Japanese sample showed 17.5% difference from that of a Malaysian sample reported in Kamimura et al. (2023). Accordingly, these two samples were placed remotely in the ML barcode tree (green characters, Fig. 1). This species was originally described as *Forficesilacurvicauda* by Motschulsky (1863) and is considered a cosmopolitan of tropical and subtropical regions of both the Old and New Worlds (Brindle 1969a, Steinmann 1990, Sakai 1993, Srivastava 2013, Nishikawa 2016, Nishikawa 2020a). Including *Forficesiladilaticauda* (Motschulsky, 1863), which was synonymised with *P.curvicauda* by Burr (1911), this species is known to be highly variable in the body colour, shape of male forceps and vestiture (Burr 1911, Borelli 1928, Hebard 1933b, Hincks 1947, Brindle 1971, Brindle 1973). Specimens analysed in Kamimura et al. (2023) and the present study (Fig. 3) may represent brown and black morphs, respectively. These two morphs also differ in the size of the male and female genitalia (YK, unpublished data), requiring further study.

Although reconstructed to be monophyletic, our analysis revealed that *Labidurariparia* (Labiduridae) has high sequence variation (14.4% at maximum; Figs 1, 2D), resulting in the formation of two major clades (D-a and D-b), which are subdivided into D-a1 and D-a2 and D-b1 and D-b2, respectively (Fig. 2D: all with > 90% bootstrap support). Clade D-a consists of the East and Southeast Asian samples, amongst which two individuals from Shikoku (temperate Japan) are classified in D-a1 and one from Ishigaki Island, Ryukyus (subtropical Japan) forming Clade D-a2 together with a Malaysian sample. Clades D-b1 and D-b2 make up clusters of samples from USA + Egypt and Europe to the Middle East, respectively. Although we tentatively treated all of these samples under the name *Ld.riparia* in this study, some previous authors have treated East, South and Southeast Asia and New Guinea populations as a distinct species, *Labidurajaponica* (de Haan, 1842), based on differences in external morphology (Burr 1911, Steinmann 1979, Steinmann 1989a, Steinmann 1989b). Further investigation of reproductive isolation amongst the clades using more samples from various localities will contribute to differentiating this possible species complex. In marked contrast to *Ld.riparia*, *Labiaminor*, another cosmopolitan species with well-developed wings, showed extremely low sequence diversity worldwide (Figs 1, 2). Surprisingly, pairwise comparisons revealed no sequence differences (0% divergence) amongst samples collected from Greece, Malta, Australia and Japan. A sample from Norway exhibited only 0.15–0.16% divergence from the others.

**Figure 4.**
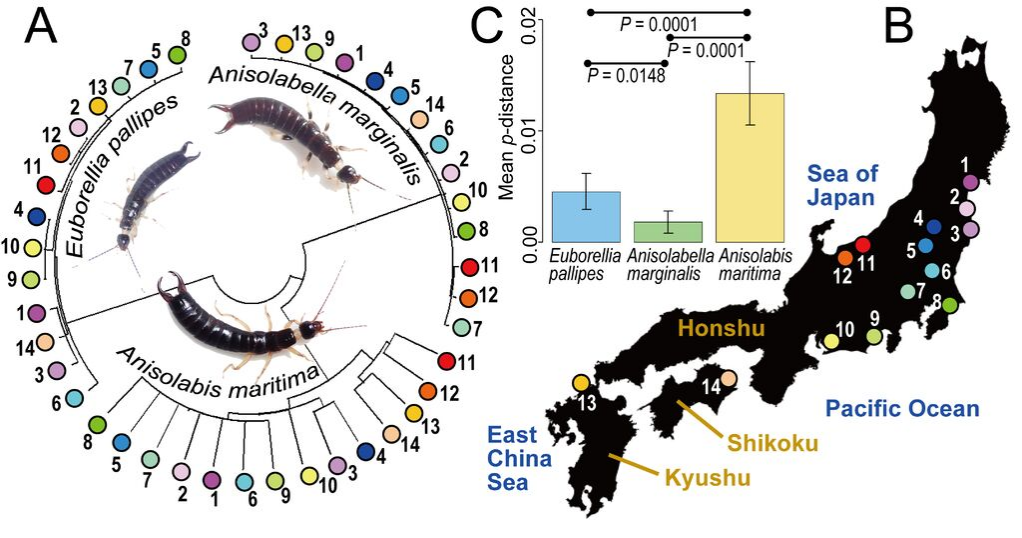
Neighbour-joining barcode tree constructed based on uncorrected genetic distances (*p*-distances) for three anisolabidid species (**A**; with male habitus [not scaled]), collected from 14 localities of Japan (**B**). Mean within-species *p*-distances are shown on the bar plot (**C**), with the standard errors estimated by 1,000-replication bootstrapping. Sampling points (1–14) are described in Table 2.

In contrast to the high within-species sequence diversity observed in *A.maritima*, the other two flightless species of Anisolabididae selected for detailed analysis, *Al.marginalis* and *E.pallipes*, showed low diversity in their barcodes (Fig. 2A and C). Observed genetic diversity can depend on the geographical sampling range; however, after selecting only 14 sampling sites, from which all three species were collected, *A.maritima* still showed significantly higher sequence diversity (average *p*-distance of 1.34%) compared with *Al.marginalis* (0.18%; 10,000 replication permutation test, *P* = 0.0001) and *E.pallipes* (0.45%; *P* = 0.0001) (Fig. 4). Sequence diversity was notably low in *Al.marginalis*, significantly lower than even that of *E.pallipes* (*P* = 0.0148). Though these statistical results must be interpreted with caution because of the interdependence of distance data, the standard errors calculated directly by bootstrapping the sequence data led to a similar conclusion (Fig. 4C).

Although *E.pallipes* has long been confused with *E.plebeja*, a fully-winged species (see Kamimura et al. (2023) for details), these three anisolabidid species are considered completely flightless (Sakai 1970, Sakai 1987, Nishikawa 2016). These semi-synanthropic species are also similar in their habitat preferences: common on the seaside, riversides, crop fields and urbanised areas in Japan (Furukawa 1965, Nishikawa 1975, Nishikawa 2016, Yamasaki 1999, Kamimura 2005). Therefore, it remains unclear why *A.maritima* shows notably higher sequence diversity than *Al.marginalis* and *E.plebeja*. Aside from the possible effects of introgression with *A.seirokui*, their wider distribution and abundance in seashore environments may be responsible for these differences. *Anisolabiismaritima* appears to be highly tolerant to high salinity (Bennet 1904) and is usually found in environments nearer to shorelines than *Al.marginalis* and *E.pallipes*. By contrast, *Al.marginalis* prefers somewhat more humid, shaded environments (Kamimura 2005). Although flightless, *A.maritima* and its allies, abundant in maritime environments, are known to readily colonise volcanic islands, possibly by debris rafting and have been found on many oceanic islands far from continents (Kevan 1965, Brindle 1968, Brindle 1969a, Brindle 1969b, Brindle 1972, Brindle 1980, Kevan and Vickery 1997, Abe 2006, Mori et al. 2020, Nishikawa 2020b). In addition, Buckel (1929) speculated on the artificial introduction of *A.maritima* to Vancouver Island, British Columbia: “They were probably introduced to Departure Bay by Japanese fishing boats, as there is a considerable trade in herrings from this point to the Orient”.

Interestingly, Kamimura et al. (2023) revealed high sequence divergence (up to 5.5%) in *E.annulata*, another circumtropical cosmopolitan that is predominantly found in seaside environments. Both *Ld.riparia* and *E.annulipes* exhibit very high sequence diversity, possibly corresponding to a complex of several cryptic species (Figs 1, 2D). The former species is also an inhabitant of open lands, especially the seaside and frequently invades new habitats (Brindle 1969a, Simberloff and Wilson 1969, Ball and Glucksman 1975, Langston and Powell 1975, Thornton et al. 2001). *Labidurariparia* is also frequently intercepted in quarantine plant inspections (personal communication by Gurney A.B. 1958, cited in Schlinger et al. (1959)). *Euborelliaannulipes* is also frequent in maritime environments and has been recorded from almost all islands in the tropics or subtropics, including oceanic archipelagos (Hebard 1933a, Hebard 1933b, Kevan 1965, Brindle 1968, Brindle 1969a, Brindle 1969b, Nishikawa 1969, Brindle 1970, Brindle 1972, Brindle 1976, Brindle 1980, Kevan and Vickery 1997, Nishikawa 2020b). For *E.annulipes*, artificial transportation of living plants is also considered to contribute to their cosmopolitan distribution in warm-temperate to tropical zones worldwide and their sporadic establishment in greenhouses in colder zones (Brindle 1966, Brindle 1969a, Kocarek et al. 2015, Nishikawa 2016, Vujić et al. 2022). Interestingly, these maritime inhabitants showed notably higher sequence diversity than *L.minor* (Fig. 1), another cosmopolitan species that flies readily with its developed wings and is attracted to light sources at night (e.g. Hebard (1917), Morse (1920), Kleinow (1966), Vickery and Kevan (1983), Vickery and Kevan (1985), Nishikawa (2016)). The high ability to colonise isolated habitats, such as oceanic islands, by the maritime species may enhance their potential for local differentiation. Sporadic long-distance transportation via ocean currents and/or human activity may contribute to stir diversified populations, resulting in their higher genetic diversity, even in small areas.

## Supplementary Material

5893B23E-0AB6-5071-B502-3575A32112B6 10.3897/BDJ.11.e107001.suppl1 Supplementary material 1 Table S1. Samples analysed in the present study Data type Table Brief description Table summarising dermapteran samples used for the analysis of DNA barcodes. File: oo_853344.pdf https://binary.pensoft.net/file/853344 Yoshitaka Kamimura, Masaru Nishikawa, Junsuke Yamasako

## Figures and Tables

**Table 1.** List of dermapteran species recorded in Japan. Modified and updated from Nishikawa (2020a).

Family	Species	Reference	Barcoded
Diplatyidae	*Diplatysflavicollis* Shiraki, 1908		x
	*Paradiplatysgladiator* (Burr, 1905)**	Nishikawa and Naka (2019)	
Pygidicranidae	*Challiaimamurai* Nishikawa, 2006		x
	*Echinosomaakitai* Nishikawa, 2023	Nishikawa (2023)	
	*Parapsalisinfernalis* (Burr, 1913)		x
Anisolabididae	*Isolabisishigakiensis* Nishikawa, 2014		
	*Anisolabellamarginalis* (Dohrn, 1864)		x
	*Anisolabellaryukyuensis* (Nishikawa, 1969)		x
	*Anisolabisboninensis* Nishikawa, 2013		
	*Anisolabismaritima* (Bonelli, 1832)		x
	*Anisolabispicea* Shiraki, 1906		
	*Anisolabisseirokui* Nishikawa, 2008		x
	*Gonolabisdistincta* (Nishikawa, 1969)	Nishikawa (2021)	x
	*Gonolabismichikoae* Nishikawa, 2021	Nishikawa (2021)	x
	*Gonolabismiyatakei* Nishikawa, 2021	Nishikawa (2021)	x
	*Euborelliaannulata* (Fabricius, 1793)	Kamimura et al. (2023)	x*
	*Euborelliaannulipes* (Lucas, 1847)		x
	*Euborelliapallipes* (Shiraki, 1906)	Kamimura et al. (2023)	x
	*Euborelliaplebeja* (Dohrn, 1863)**		x*
Labiduridae	*Labidurariparia* (Pallas, 1773)		x
	*Nalalividipes* (Dufour, 1828)		x*
Spongiphoridae	*Chaetospaniahexagonalis* Nishikawa, 2006		x
	*Labiaminor* (Linnaeus, 1758)		x
	*Paralabellulacurvicauda* (Motschulsky, 1863)		x
	*Spongovostoxsakaii* Nishikawa, 2006		x
	*Maravaarachidis* (Yersin, 1860)**		x*
	*Nesogasterlewisi* (de Bormans, 1903)		x
Chelisochidae	*Proreussimulans* (Stål, 1860)		x
Forficulidae	*Eparchusyezoensis* (Matsumura et Shiraki, 1905)		x
	*Timomenuskomarowi* (Semenov, 1901)		
	*Paratimomenusflavocapitatus* (Shiraki, 1905)		
	*Anechuraharmandi* (Burr, 1904)		x
	*Anechurajaponica* (de Bormans, 1880)**		
	*Elaunonbiparticus* (Kirby, 1891)		x*
	*Forficulaauricularia* Linnaeus, 1758**		x*
	*Forficulahiromasai* Nishikawa, 1970		x
	*Forficulamikado* Burr, 1904		x
	*Forficulaparatomis* Steinmann, 1985**		
	*Forficulaplanicollis* Kirby, 1891		
	*Forficulascudderii* de Bormans, 1880		
* At least one sequence is available in the Barcode of Life Data Systems (BOLD) and/or GenBank (accessed 28 March 2023) for non-Japanese samples only. Not included in the present analysis.			
**No established populations in Japan.			

**Table 2.** Samples analysed in the present study. See supplementary materials for further details (Suppl. material 1).

Family	Species	Code in Fig. 1 or Fig. 2	Collection site* (Location no, in Fig. 4)	DDBJ/ENA/GenBank accession no. (or BOLD sample code)
Anisolabididae	*Euborelliapallipes*	2A-01	Tokushima-Ishii [Myozai dist.]	LC767857
Anisolabididae	*Euborelliapallipes*	2A-02	Hyogo-Takasago	LC715955
Anisolabididae	*Euborelliapallipes*	2A-03	Fukushima-Iwaki	LC715956
Anisolabididae	*Euborelliapallipes*	2A-04	Kagoshima-Shimokoshiki Is.	LC715957
Anisolabididae	*Euborelliapallipes*	2A-05	Niigata-Kashiwazaki (11)	LC767802
Anisolabididae	*Euborelliapallipes*	2A-06	Saitama-Kawagoe (7)	LC767827
Anisolabididae	*Euborelliapallipes*	2A-07	Fukushima-Nishigo [Nishishirakawa dist.] (5)	LC767830
Anisolabididae	*Euborelliapallipes*	2A-08	Chiba-Mobara (8)	LC767833
Anisolabididae	*Euborelliapallipes*	2A-09	Kagoshima-Shimokoshiki Is.	LC767835
Anisolabididae	*Euborelliapallipes*	2A-10	Fukuoka-Chikuzen Oshima Is. (13)	LC767851
Anisolabididae	*Euborelliapallipes*	2A-11	Hyogo-Takasago	LC767855
Anisolabididae	*Euborelliapallipes*	2A-12	Kagoshima-Shimokoshiki Is.	LC767864
Anisolabididae	*Euborelliapallipes*	2A-13	Niigata-Joetsu (12)	LC767805
Anisolabididae	*Euborelliapallipes*	2A-14	Miyagi-Watari [Watari dist.] (2)	LC767824
Anisolabididae	*Euborelliapallipes*	2A-15	Shizuoka-Suruga [Shizuoka city] (9)	LC767817
Anisolabididae	*Euborelliapallipes*	2A-16	Aichi-Gamagori (10)	LC767868
Anisolabididae	*Euborelliapallipes*	2A-17	Fukushima-Yabuki [Yabuki dist.] (4)	LC767808
Anisolabididae	*Euborelliapallipes*	2A-18	Shizuoka-Aoi [Shizuoka city]	LC715958
Anisolabididae	*Euborelliapallipes*	2A-19	Tokushima-Tokushima	LC767837
Anisolabididae	*Euborelliapallipes*	2A-20	Tokushima-Aizumi [Itano dist.] (14)	LC767848
Anisolabididae	*Euborelliapallipes*	2A-21	Fukushima-Hirono [Futaba dist.] (3)	LC767821
Anisolabididae	*Euborelliapallipes*	2A-22	Miyagi-Miyagino [Sendai city] (1)	LC767814
Anisolabididae	*Euborelliapallipes*	2A-23	Ibaraki-Chikusei (6)	LC767811
Anisolabididae	*Euborelliaannulipes*	1G-01	Kagoshima-Amami Oshima Is.	LC731318
Anisolabididae	*Euborelliaannulipes*	1G-02	USA-Texas-Comal	HM385638
Anisolabididae	*Euborelliaannulipes*	1G-03	INDIA-Karnataka-Shivamogga	OP445698
Anisolabididae	*Gonolabismiyatakei*	1G-04	Kagoshima-Amami Oshima Is.	LC715976
Anisolabididae	*Gonolabismichikoae*	1G-05	Kagoshima-Amami Oshima Is.	LC715991
Anisolabididae	*Anisolabismaritima*	2B-01	Kagoshima-Amami Oshima Is.	LC767852
Anisolabididae	*Anisolabismaritima*	2B-02	USA-New York-Shirley	MF468287
Anisolabididae	*Anisolabismaritima*	2B-03	USA-New York-Shirley	MF468289
Anisolabididae	*Anisolabismaritima*	2B-04	USA-New York-Shirley	MF140524
Anisolabididae	*Anisolabismaritima*	2B-05	USA-New York-Calverton	MT192775
Anisolabididae	*Anisolabismaritima*	2B-06	Hokkaido-Otaru	LC767853
Anisolabididae	*Anisolabismaritima*	2B-07	Niigata-Kashiwazaki (11)	LC767804
Anisolabididae	*Anisolabismaritima*	2B-08	Niigata-Joetsu (12)	LC767807
Anisolabididae	*Anisolabisseirokui*	2B-09	Tokushima-Ooge Is.	LC715961
Anisolabididae	*Anisolabismaritima*	2B-10	Tokushima-Ooge Is.	LC715960
Anisolabididae	*Anisolabismaritima*	2B-11	Tokushima-Naruto	LC767836
Anisolabididae	*Anisolabismaritima*	2B-12	Tokushima-Naruto	LC767838
Anisolabididae	*Anisolabismaritima*	2B-13	Tokushima-Naruto (14)	LC767847
Anisolabididae	*Anisolabismaritima*	2B-14	Fukuoka-Chikuzen Oshima Is. (13)	LC767861
Anisolabididae	*Anisolabismaritima*	2B-15	Ibaraki-Chikusei (6)	LC767819
Anisolabididae	*Anisolabismaritima*	2B-16	Shizuoka-Suruga [Shizuoka city] (9)	LC767813
Anisolabididae	*Anisolabismaritima*	2B-17	Miyagi-Miyagino [Sendai city] (1)	LC767816
Anisolabididae	*Anisolabismaritima*	2B-18	Miyagi-Watari [Watari dist.] (2)	LC767826
Anisolabididae	*Anisolabismaritima*	2B-19	Saitama-Kawagoe (7)	LC767829
Anisolabididae	*Anisolabismaritima*	2B-20	Fukushima-Nishigo [Nishishirakawa dist.] (5)	LC767832
Anisolabididae	*Anisolabismaritima*	2B-21	Chiba-Shirako [Chosei dist.]	LC767850
Anisolabididae	*Anisolabismaritima*	2B-22	Chiba-Shirako [Chosei dist.] (8)	LC767858
Anisolabididae	*Anisolabismaritima*	2B-23	USA-New York-Shirley	MF468288
Anisolabididae	*Anisolabismaritima*	2B-24	USA-New York-Great South Bay	MZ701626
Anisolabididae	*Anisolabismaritima*	2B-25	USA-New York-Great South Bay	MT192774
Anisolabididae	*Anisolabismaritima*	2B-26	Aichi-Gamagori (10)	LC767834
Anisolabididae	*Anisolabismaritima*	2B-27	Kochi-Toyo [Aki dist.]	LC767841
Anisolabididae	*Anisolabismaritima*	2B-28	Fukushima-Yabuki [Yabuki dist.] (4)	LC767810
Anisolabididae	*Anisolabismaritima*	2B-29	Fukushima-Hirono [Futaba dist.] (3)	LC767823
Anisolabididae	*Anisolabisseirokui*	2B-30	Tokushima-Tokushima	LC767840
Anisolabididae	*Gonolabisdistincta*	1G-06	Okinawa-Naha	LC715963
Anisolabididae	*Gonolabisdistincta*	1G-07	Okinawa-Naha	LC715982
Anisolabididae	*Anisolabellaryukyuensis*	1G-08	Okinawa-Nago	LC715962
Anisolabididae	*Anisolabellamarginalis*	2C-01	Niigata-Joetsu (12)	LC767806
Anisolabididae	*Anisolabellamarginalis*	2C-02	Saitama-Kawagoe (7)	LC767828
Anisolabididae	*Anisolabellamarginalis*	2C-03	Niigata-Kashiwazaki (11)	LC767803
Anisolabididae	*Anisolabellamarginalis*	2C-04	Fukushima-Hirono [Futaba dist.] (3)	LC767822
Anisolabididae	*Anisolabellamarginalis*	2C-05	Fukuoka-Chikuzen Oshima Is. (13)	LC767862
Anisolabididae	*Anisolabellamarginalis*	2C-06	Shizuoka-Suruga [Shizuoka city] (9)	LC767818
Anisolabididae	*Anisolabellamarginalis*	2C-07	Miyagi-Miyagino [Sendai city] (1)	LC767815
Anisolabididae	*Anisolabellamarginalis*	2C-08	Fukushima-Yabuki [Yabuki dist.] (4)	LC767809
Anisolabididae	*Anisolabellamarginalis*	2C-09	Kanagawa-Midori [Yokohama city]	LC715980
Anisolabididae	*Anisolabellamarginalis*	2C-10	Tokushima-Aizumi [Itano dist.] (14)	LC767849
Anisolabididae	*Anisolabellamarginalis*	2C-11	Tokushima-Aizumi [Itano dist.]	LC767856
Anisolabididae	*Anisolabellamarginalis*	2C-12	SOUTH KOREA-Jeollanam do-Yeosu si	OL663261
Anisolabididae	*Anisolabellamarginalis*	2C-13	SOUTH KOREA-Jeollanam do-Yeosu si	OL663262
Anisolabididae	*Anisolabellamarginalis*	2C-14	SOUTH KOREA-Jeollanam do-Yeosu si	OL663260
Anisolabididae	*Anisolabellamarginalis*	2C-15	Fukushima-Nishigo [Nishishirakawa dist.] (5)	LC767831
Anisolabididae	*Anisolabellamarginalis*	2C-16	Tokyo-Komae	LC715985
Anisolabididae	*Anisolabellamarginalis*	2C-17	Ibaraki-Chikusei (6)	LC767812
Anisolabididae	*Anisolabellamarginalis*	2C-18	Miyagi-Watari [Watari dist.] (2)	LC767825
Anisolabididae	*Anisolabellamarginalis*	2C-19	Chiba-Oamishirasato (8)	LC767859
Anisolabididae	*Anisolabellamarginalis*	2C-20	Aichi-Gamagori (10)	LC767867
Spongiphoridae	*Paralabellulacurvicauda*	1G-09	Okinawa-Nago	LC767865
Labiduridae	*Labidurariparia*	2D-01	Ehime-Iyo	LC715965
Labiduridae	*Labidurariparia*	2D-02	Tokushima-Ishii [Myozai dist.]	LC767869
Labiduridae	*Labidurariparia*	2D-03	Okinawa-Ishigaki Is.	LC767845
Labiduridae	*Labidurariparia*	2D-04	MALAYSIA-Penang Is.	LC715964
Labiduridae	*Labidurariparia*	2D-05	USA-California-Imperial	HM376335
Labiduridae	*Labidurariparia*	2D-06	USA-California-Imperial	HM376336
Labiduridae	*Labidurariparia*	2D-07	USA-California-San Diego	(UNI37133-17)
Labiduridae	*Labidurariparia*	2D-08	USA-California-Imperial	HM376334
Labiduridae	*Labidurariparia*	2D-09	EGYPT-Alexandria-Mariot	(GMEGG330-14)
Labiduridae	*Labidurariparia*	2D-10	EGYPT-Alexandria-Mariot	(GMEGS343-14)
Labiduridae	*Labidurariparia*	2D-11	EGYPT-Alexandria-Mariot	(GMEGP026-14)
Labiduridae	*Labidurariparia*	2D-12	EGYPT-Alexandria-Mariot	(GMEGQ060-14)
Labiduridae	*Labidurariparia*	2D-13	EGYPT-Alexandria-Mariot	(GMEGP025-14)
Labiduridae	*Labidurariparia*	2D-14	EGYPT-Alexandria-Mariot	(GMEGP281-14)
Labiduridae	*Labidurariparia*	2D-15	PORTUGAL-Coimbra-Figueira da Foz	MT762852
Labiduridae	*Labidurariparia*	2D-16	PORTUGAL-Castelo Branco-Idanha a Nova	MT762853
Labiduridae	*Labidurariparia*	2D-17	PORTUGAL-Setubal-Sines	MT762842
Labiduridae	*Labidurariparia*	2D-18	GEORGIA-Kakheti-Mt. Gareja	(FHDER106-16)
Labiduridae	*Labidurariparia*	2D-19	PORTUGAL-Castelo Branco-Idanha a Nova	MT762856
Labiduridae	*Labidurariparia*	2D-20	IRAN	JN241998
Labiduridae	*Labidurariparia*	2D-21	NORTH MACEDONIA-Valandovo	(FHDER086-16)
Labiduridae	*Labidurariparia*	2D-22	ALBANIA-Gjirokaster	(FHDER114-16)
Labiduridae	*Labidurariparia*	2D-23	BULGARIA-Sozopol	(FHDER065-16)
Labiduridae	*Labidurariparia*	2D-24	BULGARIA-Montana	(FHDER062-16)
Labiduridae	*Labidurariparia*	2D-25	BULGARIA-Montana	(FHDER061-16)
Labiduridae	*Labidurariparia*	2D-26	BULGARIA-Montana	(FHDER063-16)
Labiduridae	*Labidurariparia*	2D-27	BULGARIA-Montana	(FHDER064-16)
Spongiphoridae	*Nesogasterlewisi*	1G-10	Kanagawa-Midori [Sagamihara city]	LC715986
Spongiphoridae	*Spongovostoxsakaii*	1G-11	Kagoshima-Amami Oshima Is.	LC767844
Spongiphoridae	*Chaetospaniahexagonalis*	1G-12	Okinawa-Nago	LC767820
Forficulidae	*Anechuraharmandi*	1G-13	Akita-Kazuno	LC715973
Forficulidae	*Anechuraharmandi*	1G-14	Akita-Kitaakita	LC767860
Forficulidae	*Anechuraharmandi*	1G-15	Akita-Kitaakita	LC767871
Forficulidae	*Anechuraharmandi*	1G-16	Oita-Yufu	LC767854
Forficulidae	*Anechuraharmandi*	1G-17	Ehime-Matsuyama	LC767842
Forficulidae	*Anechuraharmandi*	1G-18	Ehime-Matsuyama	LC767846
Pygidicranidae	*Challiaimamurai*	1G-19	Kagoshima-Yakushima Is.	LC715970
Pygidicranidae	*Challiaimamurai*	1G-20	Kagoshima-Yakushima Is.	LC767872
Spongiphoridae	*Paralabellulacurvicauda*	1G-21	MALAYSIA-Penang-Bukit Mertajam	LC715972
Spongiphoridae	*Labiaminor*	1G-22	GREECE-Agrafa	(FHDER131-16)
Spongiphoridae	*Labiaminor*	1G-23	AUSTRALIA-Western Australia-Perth	(WALPC3454-15)
Spongiphoridae	*Labiaminor*	1G-24	REPUBLIC OF MALTA	MW323460
Spongiphoridae	*Labiaminor*	1G-25	NORWAY-Vestfold-Stokke	(NOORT063-12)
Spongiphoridae	*Labiaminor*	1G-26	REPUBLIC OF MALTA	MW323457
Spongiphoridae	*Labiaminor*	1G-27	Hyogo-Sanda	LC767866
Forficulidae	*Forficulahiromasai*	1G-28	Kagoshima-Amami Oshima Is.	LC767843
Forficulidae	*Forficulahiromasai*	1G-29	Kagoshima-Amami Oshima Is.	LC767870
Chelisochidae	*Proreussimulans*	1G-30	Kagoshima-Tokunoshima Is.	LC767873
Chelisochidae	*Proreussimulans*	1G-31	SOUTH KOREA-detected in quarantine inspection	MW085283
Forficulidae	*Eparchusyezoensis*	1G-32	Fukushima-Minamiaizu [Minamiaizu dist.]	LC715981
Forficulidae	*Forficulamikado*	1G-33	Hokkaido-Rishiri Is.	LC767863
Forficulidae	*Forficulamikado*	1G-34	Akita-Kazuno	LC715975
Forficulidae	*Forficulamikado*	1G-35	Gifu-Takayama	LC767839
Pygidicranidae	*Diplatysflavicollis*	1G-36	Okinawa-Iriomote Is.	LC715984
Pygidicranidae	*Parapsalisinfernalis*	1G-37	Okinawa-Nago	LC715974
Pygidicranidae	*Parapsalisinfernalis*	1G-38	Okinawa-Ogimi	LC715983
* "Prefecture-City [or Island]" are indicated for Japanese samples. See main text for further details.				

**Table 3.** Intraspecific *p*-distances for the samples collected in Japan, with the average interspecific distance to the nearest species.

Family	Species	Number of Japanese samples analysed	Intraspecific distance: Minimum–Maximum	Nearest neighbour	Average distance to nearest neighbour
Diplatyidae	*Diplatysflavicollis*	1	Not available	*Spongovostoxsakaii*	0.1535
Pygidicranidae	*Challiaimamurai*	2	0.0000	*Diplatysflavicollis*	0.1634
	*Parapsalisinfernalis*	2	0.0046	*Diplatysflavicollis*	0.1672
Anisolabididae	*Anisolabellamarginalis*	17	0.0000–0.0046	*Anisolabellaryukyuensis*	0.1208
	*Anisolabellaryukyuensis*	1	Not available	*Anisolabellamarginalis*	0.1208
	*Anisolabis* spp.*	23	0.0000–0.0274	*Gonolabisdistincta*	0.1454
	*Gonolabisdistincta*	2	0.0091	*Anisolabis* spp.*	0.1454
	*Gonolabismichikoae*	1	Not available	*Gonolabismiyatakei*	0.0988
	*Gonolabismiyatakei*	1	Not available	*Gonolabismichikoae*	0.0988
	*Euborelliaannulipes*	1	Not available	*Gonolabismichikoae*	0.1709
	*Euborelliapallipes*	23	0.0000–0.0091	*Anisolabis* spp.*	0.1595
Labiduridae	*Labidurariparia*	3	0.0077–0.0756	*Spongovostoxsakaii*	0.1528
Spongiphoridae	*Chaetospaniahexagonalis*	1	Not available	*Nesogasterlewisi*	0.2006
	*Labiaminor*	1	Not available	*Forficulahiromasai*	0.1905
	*Paralabellulacurvicauda*	1	Not available	*Spongovostoxsakaii*	0.1505
	*Spongovostoxsakaii*	1	Not available	*Nesogasterlewisi*	0.1383
	*Nesogasterlewisi*	1	Not available	*Spongovostoxsakaii*	0.1383
Chelisochidae	*Proreussimulans*	1	Not available	*Eparchusyezoensis*	0.2000
Forficulidae	*Eparchusyezoensis*	1	Not available	*Proreussimulans*	0.2000
	*Anechuraharmandi*	6	0.0000–0.0243	*Challiaimamurai*	0.1995
	*Forficulahiromasai*	2	0.0243	*Nesogasterlewisi*	0.1834
	*Forficulamikado*	3	0.0106–0.0319	*Forficulahiromasai*	0.1889
* *Anisolabismaritima* and *Anisolabisseirokui*					
