# Supplementary material for: DNA barcoding of Japanese earwig species (Insecta, Dermaptera), with sequence diversity analyses of three species of Anisolabididae
Source: Biodivers Data J. 2023 Sep 27;11:e107001. doi: 10.3897/BDJ.11.e107001 (PMC10840520; doi:10.3897/BDJ.11.e107001)
Supplement: Supplementary material 1 — Table S1. Samples analysed in the present study [file bdj-11-e107001-s001.pdf]

Supplementary material 1: TableS1. Samples analyzed in the present study.

Family	Species	Code in Fig. 1 or Fig. 2	Collection site* (Location no, in Fig. 4)	Latitude, Longitude**	Collection date	Collector	Sample state	DDBJ/ENA/GenBank accession no.	Other specimen codes
Anisolabididae	*Euborellia pallipes*	2A-01	Tokushima-Ishii [Myozai dist.]	34.10N, 134.44E	9. V. 2019	Masaru Nishikawa	female	LC767857	NARO-Dermaptera-00080
Anisolabididae	*Euborellia pallipes*	2A-02	Hyogo-Takasago	Reported in Kamimura et al. (2023)				LC715955	2021BCDNA01
Anisolabididae	*Euborellia pallipes*	2A-03	Fukushima-Iwaki	Reported in Kamimura et al. (2023)				LC715956	2021BCDNA02
Anisolabididae	*Euborellia pallipes*	2A-04	Kagoshima-Shimokoshiki Is.	Reported in Kamimura et al. (2023)				LC715957	2021BCDNA03
Anisolabididae	*Euborellia pallipes*	2A-05	Niigata-Kashiwazaki (11)	37.36N, 138.56E	26. VI. 2022***	Yoshitaka Kamimura	male	LC767802	2021BCDNA112
Anisolabididae	*Euborellia pallipes*	2A-06	Saitama-Kawagoe (7)	35.91N, 139.41E	30. VIII. 2022	Yoshitaka Kamimura	female	LC767827	2021BCDNA101
Anisolabididae	*Euborellia pallipes*	2A-07	Fukushima-Nishigo [Nishishirakawa dist.] (5)	37.12N, 140.19E	20. VI. 2022	Yoshitaka Kamimura	male	LC767830	2021BCDNA104
Anisolabididae	*Euborellia pallipes*	2A-08	Chiba-Mobara (8)	35.45N, 140.30E	27. VII. 2022	Yoshitaka Kamimura	male	LC767833	2021BCDNA107
Anisolabididae	*Euborellia pallipes*	2A-09	Kagoshima-Shimokoshiki Is.	31.66N, 129.72E	19. IV. 2019	Masaru Nishikawa	male	LC767835	NARO-Dermaptera-00098
Anisolabididae	*Euborellia pallipes*	2A-10	Fukuoka-Chikuzen Oshima Is. (13)	33.90N, 130.42E	29 V. 2019	Seiji Morita	male	LC767851	NARO-Dermaptera-00091
Anisolabididae	*Euborellia pallipes*	2A-11	Hyogo-Takasago	34.75N, 134.81E	26. III. 2018	Masaru Nishikawa	female	LC767855	NARO-Dermaptera-00056
Anisolabididae	*Euborellia pallipes*	2A-12	Kagoshima-Shimokoshiki Is.	31.66N, 129.72E	19. IV. 2019	Masaru Nishikawa	male	LC767864	NARO-Dermaptera-00097
Anisolabididae	*Euborellia pallipes*	2A-13	Niigata-Joetsu (12)	37.17N, 138.24E	26. VI. 2022	Yoshitaka Kamimura	female	LC767805	2021BCDNA115
Anisolabididae	*Euborellia pallipes*	2A-14	Miyagi-Watari [Watari dist.] (2)	38.04N, 140.86E	31. V. 2022***	Yoshitaka Kamimura	male	LC767824	2021BCDNA98
Anisolabididae	*Euborellia pallipes*	2A-15	Shizuoka-Suruga [Shizuoka city] (9)	34.93N, 138.36E	16. IX. 2022	Yoshitaka Kamimura	male	LC767817	2021BCDNA88
Anisolabididae	*Euborellia pallipes*	2A-16	Aichi-Gamagori (10)	34.83N, 137.22E	28. IX. 2022	Yoshitaka Kamimura	male	LC767868	2021BCDNA111
Anisolabididae	*Euborellia pallipes*	2A-17	Fukushima-Yabuki [Yabuki dist.] (4)	37.21N, 140.33E	30. VI. 2022	Yoshitaka Kamimura	male	LC767808	2021BCDNA79
Anisolabididae	*Euborellia pallipes*	2A-18	Shizuoka-Aoi [Shizuoka city]	Reported in Kamimura et al. (2023)				LC715958	2021BCDNA64
Anisolabididae	*Euborellia pallipes*	2A-19	Tokushima-Tokushima	34.13N, 134.58E	20.V.2009***	Masaru Nishikawa	male	LC767837	NARO-Dermaptera-00011
Anisolabididae	*Euborellia pallipes*	2A-20	Tokushima-Aizumi [Itano dist.] (14)	34.14N, 134.49E	9. IV. 2019	Masaru Nishikawa	male	LC767848	NARO-Dermaptera-00070
Anisolabididae	*Euborellia pallipes*	2A-21	Fukushima-Hirono [Futaba dist.] (3)	37.21N, 141.00E	1 VIII. 2022	Yoshitaka Kamimura	male	LC767821	2021BCDNA95
Anisolabididae	*Euborellia pallipes*	2A-22	Miyagi-Miyagino [Sendai city] (1)	38.27N, 140.94E	14. VI. 2022	Yoshitaka Kamimura	male	LC767814	2021BCDNA85
Anisolabididae	*Euborellia pallipes*	2A-23	Ibaraki-Chikusei (6)	36.30N, 139.98E	16. VI. 2022	Yoshitaka Kamimura	male	LC767811	2021BCDNA82
Anisolabididae	*Euborellia annulipes*	1G-01	Kagoshima-Amami Oshima Is.	Reported in Kamimura et al. (2023)				LC731318	NARO-Dermaptera-00019
Anisolabididae	*Euborellia annulipes*	1G-02	USA-Texas-Comal	Mined from BOLD				HM385638	TTSOW187-10
Anisolabididae	*Euborellia annulipes*	1G-03	INDIA-Karnataka-Shivamogga	Mined from BOLD				OP445698	GBMNE92280-22
Anisolabididae	*Gonolabis miyatakei*	1G-04	Kagoshima-Amami Oshima Is.	Reported in Kamimura et al. (2023)				LC715976	2021BCDNA52
Anisolabididae	*Gonolabis michikoae*	1G-05	Kagoshima-Amami Oshima Is.	Reported in Kamimura et al. (2023)				LC715991	2021BCDNA72
Anisolabididae	*Anisolabis maritima*	2B-01	Kagoshima-Amami Oshima Is.	27.80N, 129.01E	9. X. 2019	Taku Tsukada	nymph	LC767852	NARO-Dermaptera-00119
Anisolabididae	*Anisolabis maritima*	2B-02	USA-New York-Shirley	Mined from BOLD				MF468287	GBMIN53020-17
Anisolabididae	*Anisolabis maritima*	2B-03	USA-New York-Shirley	Mined from BOLD				MF468289	GBMIN53020-17
Anisolabididae	*Anisolabis maritima*	2B-04	USA-New York-Shirley	Mined from BOLD				MF140524	GBMIN53022-17
Anisolabididae	*Anisolabis maritima*	2B-05	USA-New York-Calverton	Mined from BOLD				MT192775	GBMNC38357-20
Anisolabididae	*Anisolabis maritima*	2B-06	Hokkaido-Otaru	43.14N, 141.16E	22. VI. 2019	Mariko Shizuki	female	LC767853	NARO-Dermaptera-00134
Anisolabididae	*Anisolabis maritima*	2B-07	Niigata-Kashiwazaki (11)	37.36N, 138.56E	26. VI. 2022	Yoshitaka Kamimura	female	LC767804	2021BCDNA114
Anisolabididae	*Anisolabis maritima*	2B-08	Niigata-Joetsu (12)	37.17N, 138.24E	26. VI. 2022	Yoshitaka Kamimura	female	LC767807	2021BCDNA117
Anisolabididae	*Anisolabis seirokui*	2B-09	Tokushima-Ooge Is.	Reported in Kamimura et al. (2023)				LC715961	2021BCDNA12
Anisolabididae	*Anisolabis maritima*	2B-10	Tokushima-Ooge Is.	Reported in Kamimura et al. (2023)				LC715960	2021BCDNA11
Anisolabididae	*Anisolabis maritima*	2B-11	Tokushima-Naruto	34.18N,134.63E	12. V. 2019	Masaru Nishikawa	male	LC767836	NARO-Dermaptera-00100
Anisolabididae	*Anisolabis maritima*	2B-12	Tokushima-Naruto	34.18N,134.63E	12. V. 2019	Masaru Nishikawa	male	LC767838	NARO-Dermaptera-00102
Anisolabididae	*Anisolabis maritima*	2B-13	Tokushima-Naruto (14)	34.18N,134.63E	17. VI. 2018	Masaru Nishikawa	male	LC767847	NARO-Dermaptera-00052
Anisolabididae	*Anisolabis maritima*	2B-14	Fukuoka-Chikuzen Oshima Is. (13)	33.90N, 130.42E	29 V. 2019	Seiji Morita	female	LC767861	NARO-Dermaptera-00089
Anisolabididae	*Anisolabis maritima*	2B-15	Ibaraki-Chikusei (6)	36.30N, 139.98E	16. VI. 2022	Yoshitaka Kamimura	male	LC767819	2021BCDNA90
Anisolabididae	*Anisolabis maritima*	2B-16	Shizuoka-Suruga [Shizuoka city] (9)	34.93N, 138.36E	19. IX. 2022	Yoshitaka Kamimura	male	LC767813	2021BCDNA84
Anisolabididae	*Anisolabis maritima*	2B-17	Miyagi-Miyagino [Sendai city] (1)	38.27N, 140.94E	14. VI. 2022	Yoshitaka Kamimura	male	LC767816	2021BCDNA87
Anisolabididae	*Anisolabis maritima*	2B-18	Miyagi-Watari [Watari dist.] (2)	38.04N, 140.86E	31. V. 2022	Yoshitaka Kamimura	male	LC767826	2021BCDNA100
Anisolabididae	*Anisolabis maritima*	2B-19	Saitama-Kawagoe (7)	35.91N, 139.41N	30. VIII. 2022	Yoshitaka Kamimura	female	LC767829	2021BCDNA103
Anisolabididae	*Anisolabis maritima*	2B-20	Fukushima-Nishigo [Nishishirakawa dist.] (5)	37.12N, 140.19E	20. VI. 2022	Yoshitaka Kamimura	male	LC767832	2021BCDNA106
Anisolabididae	*Anisolabis maritima*	2B-21	Chiba-Shirako [Chosei dist.]	35.45N, 140.41E	28. IV. 2019	Seiji Morita	male	LC767850	NARO-Dermaptera-00083
Anisolabididae	*Anisolabis maritima*	2B-22	Chiba-Shirako [Chosei dist.] (8)	35.45N, 140.41E	28. IV. 2019	Seiji Morita	male	LC767858	NARO-Dermaptera-00082
Anisolabididae	*Anisolabis maritima*	2B-23	USA-New York-Shirley	Mined from BOLD				MF468288	GBMIN53019-17
Anisolabididae	*Anisolabis maritima*	2B-24	USA-New York-Great South Bay	Mined from BOLD				MZ701626	GBMNE21671-21
Anisolabididae	*Anisolabis maritima*	2B-25	USA-New York-Great South Bay	Mined from BOLD				MT192774	GBMNC38358-20
Anisolabididae	*Anisolabis maritima*	2B-26	Aichi-Gamagori (10)	34.83N, 137.22E	28. IX. 2022	Yoshitaka Kamimura	nymph	LC767834	2021BCDNA108
Anisolabididae	*Anisolabis maritima*	2B-27	Kochi-Toyo [Aki dist.]	33.50N, 134.26E	22. VII. 2012	Masaru Nishikawa	male	LC767841	NARO-Dermaptera-00021
Anisolabididae	*Anisolabis maritima*	2B-28	Fukushima-Yabuki [Yabuki dist.] (4)	37.21N, 140.33E	30. VI. 2022	Yoshitaka Kamimura	male	LC767810	2021BCDNA81
Anisolabididae	*Anisolabis maritima*	2B-29	Fukushima-Hirono [Futaba dist.] (3)	37.21N, 141.00E	1 VIII. 2022	Yoshitaka Kamimura	male	LC767823	2021BCDNA97
Anisolabididae	*Anisolabis seirokui*	2B-30	Tokushima-Tokushima	34.12N, 134.59E	16. VIII. 2010***	Masaru Nishikawa	male	LC767840	NARO-Dermaptera-00002
Anisolabididae	*Gonolabis distincta*	1G-06	Okinawa-Naha	Reported in Kamimura et al. (2023)				LC715963	2021BCDNA16
Anisolabididae	*Gonolabis distincta*	1G-07	Okinawa-Naha	Reported in Kamimura et al. (2023)				LC715982	2021BCDNA74
Anisolabididae	*Anisolabella ryukyuensis*	1G-08	Okinawa-Nago	Reported in Kamimura et al. (2023)				LC715962	2021BCDNA15
Anisolabididae	*Anisolabella marginalis*	2C-01	Niigata-Joetsu (12)	37.17N, 138.24E	26. VI. 2022	Yoshitaka Kamimura	male	LC767806	2021BCDNA116
Anisolabididae	*Anisolabella marginalis*	2C-02	Saitama-Kawagoe (7)	35.91N, 139.41E	30. VIII. 2022	Yoshitaka Kamimura	nymph	LC767828	2021BCDNA102
Anisolabididae	*Anisolabella marginalis*	2C-03	Niigata-Kashiwazaki (11)	37.36N, 138.56E	26. VI. 2022	Yoshitaka Kamimura	male	LC767803	2021BCDNA113
Anisolabididae	*Anisolabella marginalis*	2C-04	Fukushima-Hirono [Futaba dist.] (3)	37.21N, 141.00E	1 VIII. 2022	Yoshitaka Kamimura	female	LC767822	2021BCDNA96
Anisolabididae	*Anisolabella marginalis*	2C-05	Fukuoka-Chikuzen Oshima Is. (13)	33.90N, 130.42E	29 V. 2019	Seiji Morita	female	LC767862	NARO-Dermaptera-00090
Anisolabididae	*Anisolabella marginalis*	2C-06	Shizuoka-Suruga [Shizuoka city] (9)	34.93N, 138.36E	16. IX. 2022	Yoshitaka Kamimura	nymph	LC767818	2021BCDNA89
Anisolabididae	*Anisolabella marginalis*	2C-07	Miyagi-Miyagino [Sendai city] (1)	38.27N, 140.94E	14. VI. 2022	Yoshitaka Kamimura	male	LC767815	2021BCDNA86
Anisolabididae	*Anisolabella marginalis*	2C-08	Fukushima-Yabuki [Yabuki dist.] (4)	37.21N, 140.33E	30. VI. 2022	Yoshitaka Kamimura	male	LC767809	2021BCDNA80
Anisolabididae	*Anisolabella marginalis*	2C-09	Kanagawa-Midori [Yokohama city]	Reported in Kamimura et al. (2023)				LC715980	2021BCDNA66
Anisolabididae	*Anisolabella marginalis*	2C-10	Tokushima-Aizumi [Itano dist.] (14)	34.14N, 134.49E	1. V. 2019	Masaru Nishikawa	male	LC767849	NARO-Dermaptera-00072
Anisolabididae	*Anisolabella marginalis*	2C-11	Tokushima-Aizumi [Itano dist.]	34.14N, 134.49E	8. IV. 2019	Masaru Nishikawa	female	LC767856	NARO-Dermaptera-00069
Anisolabididae	*Anisolabella marginalis*	2C-12	SOUTH KOREA-Jeollanam do-Yeosu si	Mined from BOLD				OL663261	GBMNF32724-22
Anisolabididae	*Anisolabella marginalis*	2C-13	SOUTH KOREA-Jeollanam do-Yeosu si	Mined from BOLD				OL663262	GBMNF32725-22
Anisolabididae	*Anisolabella marginalis*	2C-14	SOUTH KOREA-Jeollanam do-Yeosu si	Mined from BOLD				OL663260	GBMNF32723-22
Anisolabididae	*Anisolabella marginalis*	2C-15	Fukushima-Nishigo [Nishishirakawa dist.] (5)	37.12N, 140.19E	20. VI. 2022	Yoshitaka Kamimura	female	LC767831	2021BCDNA105
Anisolabididae	*Anisolabella marginalis*	2C-16	Tokyo-Komae	Reported in Kamimura et al. (2023)				LC715985	2021BCDNA77
Anisolabididae	*Anisolabella marginalis*	2C-17	Ibaraki-Chikusei (6)	36.30N, 139.98E	16. VI. 2022	Yoshitaka Kamimura	male	LC767812	2021BCDNA83
Anisolabididae	*Anisolabella marginalis*	2C-18	Miyagi-Watari [Watari dist.] (2)	38.04N, 140.86E	31. V. 2022	Yoshitaka Kamimura	female	LC767825	2021BCDNA99
Anisolabididae	*Anisolabella marginalis*	2C-19	Chiba-Oamishirasato (8)	35.49N, 140.38E	28. IV. 2019	Seiji Morita	nymph	LC767859	NARO-Dermaptera-00085
Anisolabididae	*Anisolabella marginalis*	2C-20	Aichi-Gamagori (10)	34.83N, 137.22E	28. IX. 2022	Yoshitaka Kamimura	nymph	LC767867	2021BCDNA109
Spongiphoridae	*Paralabellula curvicauda*	1G-09	Okinawa-Nago	26.58N, 128.03E	21. XI. 2021***	Yoshitaka Kamimura	male	LC767865	2021BCDNA57
Labiduridae	*Labidura riparia*	2D-01	Ehime-Iyo	33.76N, 132.73E	13. VII. 2021	Yoshitaka Kamimura	male	LC715965	2021BCDNA20
Labiduridae	*Labidura riparia*	2D-02	Tokushima-Ishii [Myozai dist.]	34.10N, 134.44E	7. V. 2019	Masaru Nishikawa	male	LC767869	NARO-Dermaptera-00075
Labiduridae	*Labidura riparia*	2D-03	Okinawa-Ishigaki Is.	24.37N, 124.17E	16. VII. 2017	Tatsuo Hanatani	male	LC767845	NARO-Dermaptera-00044
Labiduridae	*Labidura riparia*	2D-04	MALAYSIA-Penang Is.	Reported in Kamimura et al. (2023)				LC715964	2021BCDNA19
Labiduridae	*Labidura riparia*	2D-05	USA-California-Imperial	Mined from BOLD				HM376335	FHDER017-09
Labiduridae	*Labidura riparia*	2D-06	USA-California-Imperial	Mined from BOLD				HM376336	FHDER018-09
Labiduridae	*Labidura riparia*	2D-07	USA-California-San Diego	Mined from BOLD					UNI37133-17
Labiduridae	*Labidura riparia*	2D-08	USA-California-Imperial	Mined from BOLD				HM376334	FHDER016-09
Labiduridae	*Labidura riparia*	2D-09	EGYPT-Alexandria-Mariot	Mined from BOLD					GMEGG330-14
Labiduridae	*Labidura riparia*	2D-10	EGYPT-Alexandria-Mariot	Mined from BOLD					GMEGS343-14
Labiduridae	*Labidura riparia*	2D-11	EGYPT-Alexandria-Mariot	Mined from BOLD					GMEGP026-14
Labiduridae	*Labidura riparia*	2D-12	EGYPT-Alexandria-Mariot	Mined from BOLD					GMEGQ060-14
Labiduridae	*Labidura riparia*	2D-13	EGYPT-Alexandria-Mariot	Mined from BOLD					GMEGP025-14
Labiduridae	*Labidura riparia*	2D-14	EGYPT-Alexandria-Mariot	Mined from BOLD					GMEGP281-14
Labiduridae	*Labidura riparia*	2D-15	PORTUGAL-Coimbra-Figueira da Foz	Mined from BOLD				MT762852	IBIDR002-20
Labiduridae	*Labidura riparia*	2D-16	PORTUGAL-Castelo Branco-Idanha a Nova	Mined from BOLD				MT762853	IBIDR005-20
Labiduridae	*Labidura riparia*	2D-17	PORTUGAL-Setubal-Sines	Mined from BOLD				MT762842	IBIDR015-20
Labiduridae	*Labidura riparia*	2D-18	GEORGIA-Kakheti-Mt. Gareja	Mined from BOLD					FHDER106-16
Labiduridae	*Labidura riparia*	2D-19	PORTUGAL-Castelo Branco-Idanha a Nova	Mined from BOLD				MT762856	IBIDR006-20
Labiduridae	*Labidura riparia*	2D-20	IRAN	Mined from BOLD				JN241998	GBA8702-12
Labiduridae	*Labidura riparia*	2D-21	NORTH MACEDONIA-Valandovo	Mined from BOLD					FHDER086-16
Labiduridae	*Labidura riparia*	2D-22	ALBANIA-Gjirokaster	Mined from BOLD					FHDER114-16
Labiduridae	*Labidura riparia*	2D-23	BULGARIA-Sozopol	Mined from BOLD					FHDER065-16
Labiduridae	*Labidura riparia*	2D-24	BULGARIA-Montana	Mined from BOLD					FHDER062-16
Labiduridae	*Labidura riparia*	2D-25	BULGARIA-Montana	Mined from BOLD					FHDER061-16
Labiduridae	*Labidura riparia*	2D-26	BULGARIA-Montana	Mined from BOLD					FHDER063-16
Labiduridae	*Labidura riparia*	2D-27	BULGARIA-Montana	Mined from BOLD					FHDER064-16
Spongiphoridae	*Nesogaster lewisi*	1G-10	Kanagawa-Midori [Sagamihara city]	35.61N, 139.20E	26. VI. 1999	Yoshitaka Kamimura	male	LC715986	2021BCDNA78
Spongiphoridae	*Spongovostox sakaii*	1G-11	Kagoshima-Amami Oshima Is.	Not available	24. V. 2018	Masaru Nishikawa	male	LC767844	NARO-Dermaptera-00041
Spongiphoridae	*Chaetospania hexagonalis*	1G-12	Okinawa-Nago	26.58N, 128.03E	23. XI. 2021***	Yoshitaka Kamimura	male	LC767820	2021BCDNA94
Forficulidae	*Anechura harmandi*	1G-13	Akita-Kazuno	40.11N, 140.84E	16-17. IX. 2019	Yoshitaka Kamimura	male	LC715973	2021BCDNA43
Forficulidae	*Anechura harmandi*	1G-14	Akita-Kitaakita	Not available	13. IV. 2019	Seiji Morita	female	LC767860	NARO-Dermaptera-00088
Forficulidae	*Anechura harmandi*	1G-15	Akita-Kitaakita	Not available	13. IV. 2019	Seiji Morita	female	LC767871	NARO-Dermaptera-00087
Forficulidae	*Anechura harmandi*	1G-16	Oita-Yufu	Not available	9. VI. 2018	Takeshi Miyake	female	LC767854	NARO-Dermaptera-00046
Forficulidae	*Anechura harmandi*	1G-17	Ehime-Matsuyama	Not available	8. VII. 2017****	Masaru Nishikawa	male	LC767842	NARO-Dermaptera-00035
Forficulidae	*Anechura harmandi*	1G-18	Ehime-Matsuyama	33.92N, 132.90E	8. VII. 2018****	Masaru Nishikawa	male	LC767846	NARO-Dermaptera-00047
Pygidicranidae	*Challia imamurai*	1G-19	Kagoshima-Yakushima Is.	30.29N, 130.41E	26. X. 2018	Taku Tsukada	male	LC715970	2021BCDNA32
Pygidicranidae	*Challia imamurai*	1G-20	Kagoshima-Yakushima Is.	Not available	26. X. 2018	Taku Tsukada	male	LC767872	NARO-Dermaptera-00065
Spongiphoridae	*Paralabellula curvicauda*	1G-21	MALAYSIA-Penang-Bukit Mertajam	Reported in Kamimura et al. (2023)				LC715972	2021BCDNA40
Spongiphoridae	*Labia minor*	1G-22	GREECE-Agrafa	Mined from BOLD					FHDER131-16
Spongiphoridae	*Labia minor*	1G-23	AUSTRALIA-Western Australia-Perth	Mined from BOLD					WALPC3454-15
Spongiphoridae	*Labia minor*	1G-24	REPUBLIC OF MALTA	Mined from BOLD				MW323460	GBMNF32755-22
Spongiphoridae	*Labia minor*	1G-25	NORWAY-Vestfold-Stokke	Mined from BOLD					NOORT063-12
Spongiphoridae	*Labia minor*	1G-26	REPUBLIC OF MALTA	Mined from BOLD				MW323457	GBMNF32754-22
Spongiphoridae	*Labia minor*	1G-27	Hyogo-Sanda	34.94N, 135.18E	27. IX. 2021***	Yoshitaka Kamimura	male	LC767866	2021BCDNA60
Forficulidae	*Forficula hiromasai*	1G-28	Kagoshima-Amami Oshima Is.	Not available	24. V. 2018	Masaru Nishikawa	male	LC767843	NARO-Dermaptera-00039
Forficulidae	*Forficula hiromasai*	1G-29	Kagoshima-Amami Oshima Is.	28.10N, 129.27E	22. V. 2018	Masaru Nishikawa	female	LC767870	NARO-Dermaptera-00038
Chelisochidae	*Proreus simulans*	1G-30	Kagoshima-Tokunoshima Is.	Not available	8. X. 2019	Taku Tsukada	female	LC767873	NARO-Dermaptera-00121
Chelisochidae	*Proreus simulans*	1G-31	SOUTH KOREA-detected in quarantine inspection	Mined from BOLD				MW085283	GBMNE21672-21
Forficulidae	*Eparchus yezoensis*	1G-32	Fukushima-Minamiaizu [Minamiaizu dist.]	37.14N, 139.71E	9. VIII. 1999	Fumio Hayashi	male	LC715981	2021BCDNA73
Forficulidae	*Forficula mikado*	1G-33	Hokkaido-Rishiri Is.	45.19N, 141.15E	25. VI. 2019	Masataka Yoshida	female	LC767863	NARO-Dermaptera-00092
Forficulidae	*Forficula mikado*	1G-34	Akita-Kazuno	40.11N, 140.84E	16-17. IX. 2019	Yoshitaka Kamimura	male	LC715975	2021BCDNA45
Forficulidae	*Forficula mikado*	1G-35	Gifu-Takayama	36.06N, 137.20E	16. IX. 2019	Masataka Yoshida	male	LC767839	NARO-Dermaptera-00116
Pygidicranidae	*Diplatys flavicollis*	1G-36	Okinawa-Iriomote Is.	24.40N, 123.80E	21-23. V. 1999	Shin'ichi Katada	male	LC715984	2021BCDNA76
Pygidicranidae	*Parapsalis infernalis*	1G-37	Okinawa-Nago	26.58N, 127.99E	23. XI. 2021	Yoshitaka Kamimura	male	LC715974	2021BCDNA44
Pygidicranidae	*Parapsalis infernalis*	1G-38	Okinawa-Ogimi	26.67N, 128.11E	18 I. 2003	Yoshitaka Kamimura	female	LC715983	2021BCDNA75

* "Prefecture-City [or Island]" are indicated for Japanese samples. See main text for further details.

** Precision of ± 0.01, which corresponds to ca. ± 1 km. All samples newly sequenced in this study were collected from unprotected areas.

*** Offspring (fixed during July 2012–December 2022) of a wild-caught female collected on the indicated date was used for analysis.

**** Collected as a nymph and emerged in the laboratory.
